# The Art of Designing Work: Work/Family Interface as a Mediator in the Relationship between Work Design, Burnout, and Performance

**DOI:** 10.3390/bs13120965

**Published:** 2023-11-23

**Authors:** Maria Luisa Giancaspro, Cataldo Giuliano Gemmano, Amelia Manuti

**Affiliations:** Department of Education, Psychology, Communication, University of Bari “Aldo Moro”, 70121 Bari, Italy; maria.giancaspro@uniba.it (M.L.G.); giuliano.gemmano@uniba.it (C.G.G.)

**Keywords:** work design, work performance, burnout, work/family interface

## Abstract

Because of the massive changes experienced within work contexts over the last decades, work design has received renewed attention both from scholars and practitioners interested in carefully balancing job demands with employees’ needs, aiming to boost performance. Hence, work design, meant as a strategic human resource management tool to craft job context and content, has been proven to impact on work performance and burnout. However, despite this evidence, the literature clearly explaining the paths through which work design might lead to positive or negative organizational outcomes is still scarce. To address this gap, the present study investigated the contribution of work–family interface aspects (i.e., work–family conflict and work–family enrichment) as mediators in this relationship. The participants were 160 white-collar employees, invited to fill in an online survey encompassing socio-demographical information (e.g., age, gender, education, and professional role) and individual self-report responses on the study variables (i.e., work design, work/family conflict, work/family enrichment, burnout, and work performance). The path analyses were conducted to investigate the direct and indirect relationships among constructs. The results showed that work–family conflict mediated the relationships between some work design characteristics and burnout, whereas work–family enrichment had a mediating role in the paths leading both to burnout and to work performance. The implications for research and practice were discussed with respect to an evidence-based human resource management perspective.

## 1. Introduction

Within the current occupational scenario, featuring radical and rapid changes that have impacted work processes as well as human resource management, work design (WD) has become an even more strategic tool to craft job descriptions, workload, tasks, and responsibilities, and to balance organizational demands with employees’ needs, thus enhancing well-being and performance [1]. Hence, several studies have confirmed the relationship between a poor WD and burnout [2,3,4,5], as well as between an effective WD and work performance [6,7,8,9]. However, despite the robust empirical evidence supporting the relationship between WD and organizational positive (performance) and negative (burnout) behaviors, research investigating the potential mediators of this relationship is still scarce. This gap needs to be addressed both for theoretical and practical reasons. Theoretically, because it suggests that there still is not an evidence-based foundation of “how and why” WD could be related to lower levels of burnout and/or to better performance. Therefore, there is a lack of knowledge about the underlying processes that might regulate the relationship between these variables. Practically, this gap limits any possibility to draw conclusions and to make recommendations to organizations and managers about how to drive WD as a strategic tool to produce desired outcomes.

In response to this evidence, the present study aimed to contribute to fill this gap, testing the role of some aspects of the work–family (WF) interface, namely, WF conflict and WF enrichment, as mediators in the relationship between WD, burnout, and work performance. Attention was focused on these WF interface aspects because a broad literature supports the importance of WD dimensions in promoting positive interrelationships between work and family [10], which, in turn, could impact burnout [11,12] and work performance [13,14,15]. Thus, WF conflict and WF enrichment were expected to be some of the mechanisms linking WD to the desired outcomes. Therefore, the goal of the present study was to investigate the indirect effects of WD characteristics (i.e., task, knowledge, social, and contextual characteristics) on burnout and work performance via WF interface aspects (i.e., WF conflict and WF enrichment).

By uncovering the role of WF conflict and WF enrichment as mediators through which WD is related to burnout and work performance, our study aimed at contributing to the expanding knowledge about the mechanisms underlying the relationship between WD and positive and negative organizational outcomes, which is, as said above, still scarce. From a practical perspective, the mediating role of WF interface aspects (i.e., WF conflict and WF enrichment) raises the need for organizations and managers to adopt strategies aimed at supporting the integration between work and life domains. In particular, the role of WF interface aspects in the path that leads to burnout may help organizations to understand which are the most challenging features of work, associated with tasks, contents, and organization, that could impact the ability of employees to balance competing demands coming from both domains. Whereas, their role in the path leading to work performance may help organizations to design work and to manage human resources, enhancing the aspects that are more attuned with employees’ expectations and with the empowerment of resources, which could contribute to a positive spillover and to WF enrichment. In both cases, the results coming from the present study could have practical implications for the development of people-based human resource management policies and practices.

## 2. Theoretical Background

### 2.1. Work Design, WF Conflict, and WF Enrichment

The radical changes that have overwhelmed the labor market in recent years have led scholars and practitioners to focus attention on the conditions that could best support individuals and organizations in maintaining high production standards and thriving even in a difficult scenario. In particular, the investigation of the mechanisms governing the organizational system (e.g., the organization of work and/or the conditions that might nurture employees’ well-being) is strategically used to read the signs of changes and to direct the management process. In light of the profound wider changes that have impacted the labor market (e.g., technological advancement and global competition among the main ones), to survive, organizations have been forced to redefine their working processes, consequently revising job descriptions and role requirements, rediscovering WD as a good practice of HR and organizational management. Hence, WD “concerns the content and organization of one’s work tasks, activities, relationships and responsibilities” [16] (p. 662).

Historically, the interest in WD has stemmed from the widespread application of the principles of scientific management in the design of initial industrial jobs. But, if, on the one hand, this approach aimed for a simplification of any management process, the motivational WD perspectives appear much more focused on the enhancement of the positive effects that work design might have on positive organizational behaviors. For instance, the job characteristics model [17] maintained that an effective WD should consider five core job characteristics (i.e., variety, autonomy, feedback, significance, and identity) to produce three individual positive psychological states (i.e., experiencing meaning, feeling responsible for outcomes, and understanding the results of their efforts), which, in turn, were proven to impact on positive work outcomes (e.g., intrinsic motivation, job satisfaction, and work performance). In this vein, the adequate WD of tasks, activities, relationships, and responsibilities could be highly strategic to manage human resources with respect to several organizational goals, such as safety, competitiveness, and innovation.

The contemporary perspective on WD [18] focuses on the four dimensions of work characteristics: task, knowledge, social, and work context characteristics. Task characteristics refer to the complexity of the tasks involved to accomplish the work of a particular job. In particular, they include the following specific aspects that characterize job tasks: autonomy, task significance, and feedback from the job. Knowledge characteristics refer to the individual requirements of a job in terms of knowledge, skills, and ability. They include the extent to which information processing and skill variety are required at work. Social characteristics refer to the nature of relational interactions involved in the execution of work activities. They include not only the social support that comes from the work climate, but also the specific feedback that comes from colleagues and supervisors. Work context characteristics refer to the physical and environmental aspects of a particular job. They include the physical demands required from the job and the conditions of the work environment [19,20]. Accordingly, the relationship between the person and work is often regulated by job characteristics and WD strategies also influence work–life balance. For many employees, work and family are the prevailing domains of life. The need to balance the two domains has become a part of all employees’ daily lives because of the increase of dual-earner households and non-traditional gender roles [21,22].

WF conflict is commonly defined as “a form of inter-role conflict in which the role pressures from the work and family domains are mutually incompatible in some respect” [23] (p. 77). WF conflict is interpreted in two directions: work interference with family (WIF) and family interference with work (FIW) [24]. WIF is caused by the work demands (e.g., long working hours and work overload) that compromise the family role [25,26]. FIW is caused by those family demands (e.g., responsibilities toward family members) that that have detrimental effects on the work domain [27]. Based on this theorization, empirical evidence has shown that WIF and FIW are influenced by work and family demands and they also have consequences on work and family outcomes, such as performance and satisfaction, e.g., [28,29,30]. Among the predictors of WF conflict, several studies ascertained that role demands have relevant effects on WF conflict, showing that work demands associated more with WIF than with FIW and family role demands associated more with FIW than with WIF [10,29,31]. Michel and colleagues’ [10] meta-analysis on the antecedents of WF conflict supported the relevance of work role stressors (e.g., role overload and time demands), work role involvement (e.g., job involvement and work centrality), work social support (e.g., organizational and peer support), and work characteristics (e.g., task variety and job autonomy) as predictors of WF conflict.

Given this evidence, the content and context features of the job were proven to play a crucial role in favoring a balance and reducing the conflict between work and family domains. For instance, job complexity is an enabling resource that could increase individual performance in the work domain as well as in other domains [32]. Hence, job complexity requires the development of skills and abilities (e.g., handling multiple tasks, planning, and organizing) to complete the work, which could also become useful skills to manage the WF interface. Likewise, the positive characteristics of the job (e.g., autonomy and decision control) have been proven to promote WF enrichment and decrease WF conflict through adequate work resources that sustain individual coping [10,33].

Recently, scholars have investigated the influence of the WF interface on individual attitudes and behaviors. These studies have led to the development of different constructs such as positive spillover [34,35] and WF facilitation [36,37,38] or work–family synergy [39]. Among these, WF enrichment was proven to have a relevant role in the process of WF balance [40,41,42]. Greenhaus and Powell define WF enrichment as “the extent to which experience in one role improves the quality of life namely performance or affect, in the other role” [12] (p. 6). However, further studies have largely suggested positive relationships between job characteristics and some nuances of WF enrichment, such as positive spillover [43,44] and WF facilitation [32,45]. The underlying assumption common to many of these approaches is that motivating job characteristics (e.g., autonomy, variety, and feedback) could provide successful and satisfactory work experiences, which, in turn, might contribute to facilitating the family role [17,46]. These characteristics are related to specific resources (e.g., time management skills and self-confidence) that could be applied to family activities and relationships to enrich the family domain though the work role [32].

### 2.2. WF Variables, Burnout, and Work Performance

The search for a balance between work and personal domains is a very expensive activity in terms of resources and energy for workers. Generally, the balance between work and family fosters psychological and physiological health and organizational outcomes [47]. On the other hand, the conflict between work and family roles leads to negative outcomes in both domains, such as emotional exhaustion and burnout [11,48]. Maslach defines burnout as “a psychological syndrome of emotional exhaustion, depersonalization, and reduced personal accomplishment that can occur among normal individuals who work with people in some capacity” [49] (pp. 20–21). A recent attempt to overcome this traditional conception of the construct and its related measurement tool came from a recent study by Schaufeli, De Witte, and Desart [50] who developed the Burnout Assessment Tool (BAT). The theoretical framework that inspired the authors sees burnout as a syndrome that consists of four interrelated components. The first component is exhaustion, which involves the depletion of physical and psychological resources. The second one is mental distance, which involves the indifference and disenchantment toward the meaning of work; the third is emotional impairment, which refers to overwhelming negative emotions at work; and the last one is cognitive impairment, which refers to the indicators of declined cognitive processes.

Among the several antecedents of burnout, WF conflict is considered a prominent one [48,51,52,53,54]. WF conflict drains individual mental and physical resources to face the imbalance between work and family demands [54]. Therefore, burnout could arise at work because of the mental and physical fatigue caused by the detrimental process of WF conflict. At the same time, in line with the COR theory [55], WF enrichment is negatively associated with burnout, because a resource gain spiral may be sparked by WF enrichment, producing an increase in physical and psychological resources which compensate for the difficulties of work demands [56,57]. For instance, developing new work competencies and applying them to the family domain may decrease burnout components because it sustains the family role with new resources. Moreover, the family domain may give employees additional resources (e.g., esteem, support, and flexibility) that may sustain the work role performance [12,44].

Another outcome considered in this study is represented by work performance. In contrast to job burnout, work performance is considered a positive outcome of individual and organizational health. It is difficult to provide an unequivocal definition of work performance as it is a multifaceted construct with numerous meanings: from performance assessment to proactive behavior [58], organizational citizenship behavior [59], counterproductive behavior [60], adaptive performance [61], and contextual performance [62]. The perspective proposed by Griffin and colleagues [63] was adopted in the present study. They developed a model consisting of three behavior dimensions (proficiency, adaptivity, and proactivity) at different organizational levels (individual, team, and organization). Proficiency refers to work performance in terms of the accomplishment of activities that are required for a specific job. Adaptivity refers to the extent to which employees adapt and cope with changes in the job and work environment. Proactivity refers to the extent to which employees take initiative and propose ideas to enhance work activities and contexts.

In this direction, as previously stated, WF conflict is associated with a decrease in performance. Previous research has shown that WF conflict impacts on work performance [13,14,15]. The influence of WF conflict on work performance is supported by the role theory and by the scarcity of resources hypothesis, which state that individual personal resources are limited and can be depleted by the demands of one role, such that they can also be insufficient for other roles [64,65,66]. Moreover, heavy workloads drain the individual capacity to balance different roles, leading to emotional exhaustion and poor performance at work. For the same reasons, WF enrichment is positively related to work performance [67,68]. The role accumulation [66,69] and the conservation of resources (COR) [70,71] theories support the idea that individual resources can also be expanded. Using and developing resources in one domain may bring improvement in resources that can be used in other domains. For instance, learning strategies for managing conflict at work may help individuals to use that skills in the family domain to obtain positive effects and enrich both the work and family roles.

**Figure 1:**
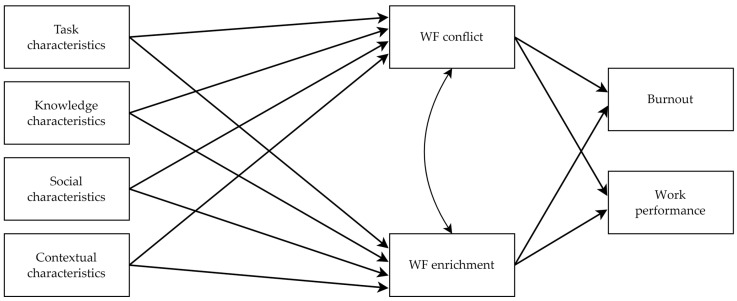
The research model hypothesizing the indirect effects of WD dimensions (i.e., task, knowledge, social, and contextual characteristics) on burnout and work performance via WF conflict and WF enrichment.

Considering the theoretical arguments and empirical evidence presented above related to WD’s influence on WF conflict and enrichment and their effects on burnout and performance, we expect that WD dimensions will have indirect effects on burnout and performance via WF conflict and WF enrichment (see Figure 1). Thus, we hypothesize the following:

**H1.**  *There is a negative indirect effect of WD dimensions (i.e., task, knowledge, social, and contextual characteristics) on burnout via WF conflict (H1a) and WF enrichment (H1b).*

**H2.**  *There is a positive indirect effect of WD dimensions (i.e., task, knowledge, social, and contextual characteristics) on work performance via WF conflict (H2a) and WF enrichment (H2b).*

## 3. Materials and Methods

### 3.1. Procedure and Sample

A power analysis was carried out to estimate the suitable sample size using the following parameters: a *p* level of 0.05; a cautious effect size of 0.15; and a power of 0.95. Results indicated that a sample size of 146 participants was adequate to warrant a 95% chance of correctly rejecting the null hypothesis. A convenience sample of 160 white-collar employees was recruited from April to May 2023. A digital call for participation was widely spread via email, social networks, and blogs to reach the network of contacts of the research team. To encourage participation, several reminders were shared on those digital platforms. A description of the research aims and the invitation to participate voluntarily and anonymously in the study was proposed to respondents. The study observed the Helsinki Declaration and the prescriptions of the General Data Protection European Regulation (EU n. 2016/679). The questionnaire gathered socio-demographical information (e.g., age, gender, education, and professional role) and individual response on the study variables (i.e., WD, WF conflict, WF enrichment, burnout, and work performance). Participants were 74% female and 26% male, with an average age of 37.96 (SD = 12.96). Regarding education, 63% had a university degree, 32% had a high school diploma, and the remaining 5% had a middle school diploma.

### 3.2. Measures

The variables included in the research model were assessed using previously validated instruments aimed at measuring WD dimensions (i.e., task, knowledge, social, and contextual characteristics), WF conflict, WF enrichment, burnout, and work performance.

#### 3.2.1. Task Characteristics

This variable was assessed using the nine items of task characteristics taken from the short version [72] of the Work Design Questionnaire [20]. Participants answered on a 5-point response scale ranging from 1, “completely disagree”, to 5, “completely agree”. This dimension of WD consists of three factors with three items each: autonomy, task significance, and feedback from the job. A sample item is “The job allows me to make a lot of decisions on my own”. A robust maximum likelihood confirmatory factor analysis (CFA) was conducted to evaluate the measurement model. It showed an adequate fit for a three-factor model (χ^2^ (22) = 53.52, *p* < 0.001, CFI = 0.95, TLI = 0.93, RMSEA = 0.09, SRMR = 0.05) after allowing the residual variances of two couples of items to be correlated as suggested by modification indices. All the standardized factor loadings were statistically significant and ranged between 0.62 and 0.87. In the present sample, Cronbach’s alphas were 0.80, 0.79, and 0.88, for autonomy, task significance, and feedback from the job, respectively. We summed up the scores of the items of the three factors to obtain a total score of task characteristics (α = 0.84).

#### 3.2.2. Knowledge Characteristics

This variable was assessed using the six items of knowledge characteristics taken from the short version [72] of the Work Design Questionnaire [20]. Participants answered on a 5-point response scale ranging from 1, “completely disagree”, to 5, “completely agree”. This dimension of WD consists of two factors with three items each: information processing and skill variety. A sample item is “The job requires that I engage in a large amount of thinking”. A CFA showed an adequate fit for a two-factor model (χ^2^ (8) = 30.82, *p* < 0.001, CFI = 0.95, TLI = 0.93, RMSEA = 0.13, SRMR = 0.04). All the standardized factor loadings were statistically significant and ranged between 0.63 and 0.89. In the present sample, Cronbach’s alphas were 0.87 for information processing and 0.81 for skill variety. We summed up the scores of the items of both factors to obtain a total score of knowledge characteristics (α = 0.89).

#### 3.2.3. Social Characteristics

This variable was assessed using the six items of social characteristics taken from the short version [72] of the Work Design Questionnaire [20]. Participants answered on a 5-point response scale ranging from 1, “completely disagree”, to 5, “completely agree”. This dimension of WD consists of two factors with three items each: social support and feedback from others. A sample item is “I receive feedback on my performance from other people in my organization”. A CFA showed an adequate fit for a two-factor model (χ^2^ (8) = 16.03, *p* < 0.05, CFI = 0.97, TLI = 0.95, RMSEA = 0.08, SRMR = 0.04). All the standardized factor loadings were statistically significant and ranged between 0.36 and 0.95. In the present sample, Cronbach’s alphas were 0.72 for social support and 0.71 for feedback from others. We summed up the scores of the items of both factors to obtain a total score of social characteristics (α = 0.77).

#### 3.2.4. Contextual Characteristics

This variable was assessed using the six items of contextual characteristics taken from the short version [72] of the Work Design Questionnaire [20]. Participants answered on a 5-point response scale ranging from 1, “completely disagree”, to 5, “completely agree”. This dimension of WD consists of two factors with three items each: physical demands and work conditions. A sample item is “The job occurs in a clean environment”. The items of physical demands were reversed to obtain a measure of contextual characteristics of low physical demands and positive work conditions. A CFA showed an adequate fit for a two-factor model (χ^2^ (8) = 9.04, *p* > 0.05, CFI = 1.00, TLI = 1.00, RMSEA = 0.03, SRMR = 0.03). All the standardized factor loadings were statistically significant and ranged between 0.56 and 0.98. In the present sample, Cronbach’s alphas were 0.92 for physical demands and 0.66 for work conditions. We summed up the scores of the items of both factors to obtain a total score of contextual characteristics (α = 0.72).

#### 3.2.5. WF Conflict

This variable was assessed using the Italian version [73] of the 10 items developed by Netemeyer and colleagues [74]. Participants answered on a 7-point response scale ranging from 1, “completely disagree”, to 7, “completely agree”. The measure consists of two factors with five items each that refer to the two directions of conflict: work-to-family and family-to-work. A sample item of the work-to-family conflict is “The demands of my work interfere with my home and family life”. A sample item of the family-to-work conflict is “The demands of my family or spouse/partner interfere with work-related activities”. A CFA showed an adequate fit for a two-factor measurement model (χ^2^ (31) = 72.29, *p* < 0.001, CFI = 0.96, TLI = 0.95, RMSEA = 0.09, SRMR = 0.07) after allowing the residual variances of three couples of items to be correlated as suggested by modification indices. All the standardized factor loadings were statistically significant and ranged between 0.60 and 0.97. In the present sample, Cronbach’s alphas were 0.91 and 0.86, for work-to-family and family-to-work conflict, respectively. We summed up the scores of the items of both dimensions to obtain a total score of WF conflict (α = 0.88).

#### 3.2.6. WF Enrichment

This variable was assessed using the Italian version [75] of the six items developed by Carlson and colleagues [42]. Participants answered on a 5-point response scale ranging from 1, “completely disagree”, to 5, “completely agree”. The measure consists of two factors with three items each that refer to the two directions of enrichment: work-to-family and family-to-work. A sample item of the work-to-family enrichment is “At work I feel positive emotions, and this helps me to be a better family member”. A sample item of the family-to-work enrichment is “In my family life I feel positive emotions, and this helps me to work better”. A CFA showed an adequate fit for a two-factor measurement model (χ^2^ (6) = 13.64, *p* < 0.05, CFI = 0.98, TLI = 0.96, RMSEA = 0.09, SRMR = 0.03) after allowing the residual variances of two couples of items to be correlated as suggested by modification indices. All the standardized factor loadings were statistically significant and ranged between 0.67 and 0.93. In the present sample, Cronbach’s alphas were 0.85 and 0.82, for work-to-family and family-to-work enrichment, respectively. We summed up the scores of the items of both dimensions to obtain a total score of WF enrichment (α = 0.87).

#### 3.2.7. Burnout

The Italian short version [76] of the Burnout Assessment Tool [50] was used to assess this variable. The instrument consists of 12 items distributed into four dimensions. Participants answered on a 5-point response scale ranging from 1, “never”, to 5, “always”. The first dimension (i.e., exhaustion) refers to physical and psychological resource depletion. A sample item is “At work, I feel mentally exhausted”. The second dimension (i.e., mental distance) refers to the disenchantment with the work. A sample item is “I struggle to find any enthusiasm for my work”. The third dimension (i.e., emotional impairment) refers to negative emotions associated with daily tasks. A sample item is “At work, I feel unable to control my emotions”. The fourth dimension (i.e., cognitive impairment) refers to signs of detriment of cognitive processes. A sample item is “At work, I have trouble staying focused”. A CFA showed an adequate fit for a four-factor measurement model (χ^2^ (48) = 65.17, *p* > 0.05, CFI = 0.98, TLI = 0.98, RMSEA = 0.05, SRMR = 0.05). All the standardized factor loadings were statistically significant and ranged between 0.63 and 0.95. In the present sample, Cronbach’s alphas were 0.87, 0.84, 0.80, and 0.83, for exhaustion, mental distance, emotional impairment, and cognitive impairment, respectively. We summed up the scores of the items of the four dimensions to obtain a total score of burnout (α = 0.89).

#### 3.2.8. Work Performance

This variable was assessed using the nine items of individual work role performance developed by Griffin and colleagues [63]. Participants answered on a 5-point response scale ranging from 1, “very little”, to 5, “a great deal”. The measure consists of three factors with three items each that refer to the three dimensions of work performance: task proficiency, task adaptivity, and task proactivity. The first dimension (i.e., task adaptivity) refers to workers’ behaviors that satisfy the known expectations and requirements of their role. A sample item is “You carried out the core parts of your job well”. The second dimension (i.e., task adaptivity) refers to behaviors of coping with changes at work. A sample item is “You adapted well to changes in core tasks”. The third dimension (i.e., task proactivity) refers to self-starting behaviors aimed at improving or innovating their own work. A sample item is “You come up with ideas to improve the way in which your core tasks are done”. A CFA showed an adequate fit for a three-factor measurement model (χ^2^ (23) = 54.85, *p* < 0.001, CFI = 0.95, TLI = 0.93, RMSEA = 0.09, SRMR = 0.05) after allowing the residual variances of one couple of items to be correlated as suggested by modification indices. All the standardized factor loadings were statistically significant and ranged between 0.60 and 0.82. In the present sample, Cronbach’s alphas were 0.77, 0.80, and 0.80 for task proficiency, task adaptivity, and task proactivity, respectively. We summed up the scores of the items of the three dimensions to obtain a total score of work performance (α = 0.88).

### 3.3. Data Analysis

Preliminary analyses were conducted to examine descriptive statistics, validity and reliability of measures, and bivariate correlations between study variables. Univariate and multivariate normality assumptions were checked. Although the observed variables had skewness and kurtosis values < |1.00|, the result of Henze and Zirkler’s [77] index was significant (1.16, *p* < 0.001), indicating that the multivariate normality assumption was not met. Therefore, the CFAs and path analysis reported here were conducted using the robust maximum likelihood (MLR) method of estimation, due to its higher robustness to nonnormality. Our hypotheses were tested by means of path analysis with observed scores. Specifically, a mediation model with four exogenous variables (WD dimensions), two parallel mediators (WF conflict and WF enrichment), and two outcomes (burnout and work performance) was fitted. A bootstrapping procedure (with 5000 bootstrapped samples) was used to compute the hypothesized indirect effects’ 95% confidence interval (CI). All analyses were conducted using the Jamovi software (https://www.jamovi.org (accessed on 19 June 2023)) [78] and the PATHj module (https://pathj.github.io (accessed on 19 June 2023)) [79].

## 4. Results

Table 1 shows the means, standard deviations, skewness, kurtosis, Cronbach’s alpha coefficients, and Pearson’s correlations associated with the study variables.

For a first examination of the direct relationships between the study variables, we fitted a just-identified model (Model 1) including all the direct effects of the four dimensions of WD (i.e., task, knowledge, social, and contextual characteristics) on WF conflict, WF enrichment, burnout, and work performance, as well as the direct effects of WF conflict and WF enrichment on burnout and work performance. The results of Model 1 showed that the task characteristics were negatively related to WF conflict (−0.44, SE = 0.15, *p* < 0.01) and positively related to WF enrichment (0.33, SE = 0.06, *p* < 0.001). The knowledge characteristics had no significant relationships with WF conflict (0.36, SE = 0.19, *p* > 0.05) and WF enrichment (−0.14, SE = 0.08, *p* > 0.05). The social characteristics had a non-significant relationship with WF conflict (0.06, SE = 0.21, *p* > 0.05) and a significant positive relationship with WF enrichment (0.32, SE = 0.10, *p* < 0.01). The contextual characteristics had a significant relationship with WF conflict (−0.70, SE = 0.20, *p* < 0.001) and a non-significant relationship with WF enrichment (0.06, SE = 0.08, *p* > 0.05). WF conflict had a significant positive relationship with burnout (0.20, SE = 0.05, *p* < 0.001) and a non-significant relationship with work performance (0.02, SE = 0.04, *p* > 0.05). WF enrichment had a significant negative relationship with burnout (−0.63, SE = 0.14, *p* < 0.001) and a significant positive relationship with work performance (0.25, SE = 0.10, *p* < 0.05). All the direct effects of the four WD dimensions on burnout and work performance were not significant, except for a significant negative relationship between task characteristics and burnout (−0.28, SE = 0.11, *p* < 0.001).

**Figure 2:**
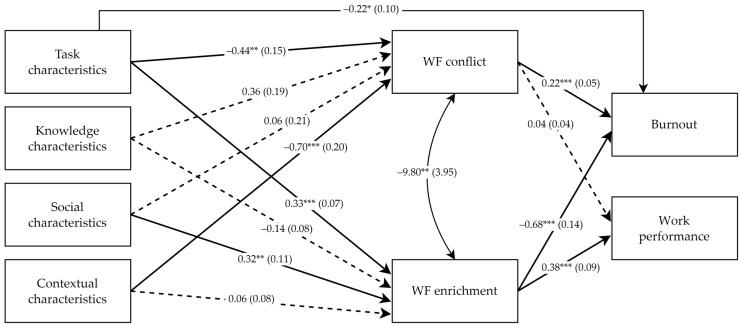
Parameter estimates for Model 2. Note. *** *p* < 0.001; ** *p* < 0.01; * *p* < 0.05. Coefficients are unstandardized. Standard errors are shown within parentheses. Dotted lines indicate non-significant relationships.

Adopting a parsimonious approach, we fitted a second model (Model 2), including the only significant direct effect of task characteristics on burnout, and constraining to zero the other effects of WD dimensions on burnout and work performance. The goodness-of-fit for Model 2 was satisfactory (χ^2^ (7) = 11.59, *p* > 0.05, CFI = 0.98, TLI = 0.93, RMSEA = 0.06, SRMR = 0.04). Figure 2 shows the unstandardized parameter estimates obtained for the involved direct effects of Model 2. The parameter estimates and statistical significance of the direct effects of Model 2 (see Figure 2) were like the results of Model 1 described above. Thus, the more parsimonious Model 2 was retained, and it was used to test the indirect effects associated with Hypotheses 1 and 2.

Table 2 shows the unstandardized parameter estimates and 95% CI of the indirect effects of Model 2, computed using a bootstrapping procedure. The indirect effects of task characteristics on burnout via WF conflict (−0.10, SE = 0.04, 95% CI = −0.20; −0.03) and via WF enrichment (−0.23, SE = 0.07, 95% CI = −0.40; −0.11) were negative and statistically significant. The indirect effect of task characteristics on work performance via WF conflict was not significant (−0.02, SE = 0.02, 95% CI = −0.06; 0.01), whereas the same effect via WF enrichment was positive and significant (0.13, SE = 0.04, 95% CI = 0.06; 0.22). All the indirect effects of the knowledge characteristics on burnout and work performance via WF conflict and WF enrichment were not statistically significant (see Table 2). The indirect effect of the social characteristics on burnout via WF conflict was not significant (0.01, SE = 0.05, 95% CI = −0.08; 0.11), whereas the same effect via WF enrichment was negative and significant (−0.22, SE = 0.08, 95% CI = −0.42; −0.09). The indirect effect of the social characteristics on work performance via WF conflict was not significant (0.00, SE = 0.01, 95% CI = −0.01; 0.04), whereas the same effect via WF enrichment was positive and significant (0.12, SE = 0.05, 95% CI = 0.04; 0.23). The indirect effect of the contextual characteristics on burnout via WF conflict was negative and significant (−0.15, SE = 0.06, 95% CI = −0.29; −0.05), whereas the same effect via WF enrichment was not significant (−0.04, SE = 0.06, 95% CI = −0.16; 0.06). Both the indirect effects of the contextual characteristics on work performance via WF conflict and WF enrichment were not statistically significant (see Table 2). The evaluation of the R^2^ shows that the model accounted for 14% of the variance in WF conflict, 36% of the variance in WF enrichment, 45% of the variance in burnout, and 13% of the variance in work performance.

## 5. Discussion and Conclusions

The present study aimed at understanding and explaining the mechanisms through which work design (WD) dimensions (i.e., task, knowledge, social, and contextual characteristics) are linked to burnout and work performance, hypothesizing the mediating roles of work–family (WF) interface aspects (i.e., WF conflict and WF enrichment). Specifically, a negative indirect relationship between WD dimensions and burnout through the mediation of WF conflict (H1a) and WF enrichment (H1b) was hypothesized. Also, a positive indirect relationship between WD dimensions and work performance through the mediation of WF conflict (H2a) and WF enrichment (H2b) was hypothesized. For this purpose, a convenience sample of 160 white-collar employees was gathered to fill in an online survey encompassing self-report responses on the study variables. Path analyses were conducted to test the hypothesized relationships. The hypotheses were partially confirmed because some, but not all, WD characteristics had significant indirect effects on the outcomes through the parallel mediations of WF conflict and WF enrichment.

Hypothesis H1a was partially confirmed because the results showed that WF conflict mediated the relationships between some WD characteristics and burnout. Specifically, the task and contextual characteristics were negatively related to WF conflict, which, in turn, was positively related to burnout. On the other hand, the knowledge and social characteristics were not significantly related to WF conflict and burnout. These relationships suggested that the task and contextual characteristics of WD may contribute to preventing burnout because they contribute to limiting the conflict between work and family domains. This evidence was in line with previous studies showing the crucial role played by organizations in buffering the negative effects of work organization on WF conflict and consequently on performance and well-being [10,14,80]. Accordingly, the conclusion drawn by most research in the field is that supporting a work–family culture (e.g., through WF programs addressed to craft job demands in line with employees’ needs) is the key to increasing well-being and performance, limiting the negative effects of burnout [81]. In this vein, the present study confirmed that WD could be, among others, a strategic tool to manage people and organizations.

Hypothesis H2a was not confirmed because the indirect effects of the WD characteristics on work performance via WF conflict were not significant. Specifically, WF conflict was not related to work performance. This non-significant relationship suggested that other aspects of the WF interface (e.g., WF enrichment) could have relevant roles in affecting employees’ performance. WF conflict could be the mediating mechanism in a negative process that leads to undesirable outcomes (e.g., burnout) but it does not contribute to reducing the work performance because other WF variables (e.g., WF enrichment) intervene in the positive process that leads to desired outcomes.

Hypotheses H1b and H2b were partially confirmed because the results showed that WF enrichment mediated the impact of some WD characteristics leads to both burnout and work performance. Specifically, the task and social characteristics were positively related to WF enrichment, which, in turn, was negatively related to burnout and positively related to work performance. On the other hand, the knowledge and contextual characteristics were not significantly related to WF enrichment. Hence, the task and social characteristics of WD may contribute to enhancing work performance and decreasing the risk of burnout because they foster the mutual enrichment between work and family roles. This evidence was in line with previous studies on positive spillover showing that if the working context is designed in a way that conveys a supportive work–family culture, then employees will be involved in sharing positive feelings of motivation and work engagement that will finally result in work–family enrichment [82].

In the tested model, the knowledge characteristics were not significantly related to WF aspects, burnout, and work performance. The non-significant role played by knowledge characteristics suggested that maybe the other characteristics of WD could be relevant in the relationship with burnout and performance, especially in the specific category of the workers (i.e., white-collar employees) involved in the present study. Hence, white-collar jobs are typically defined by the high levels of knowledge characteristics in terms of information processing and skill variety. Therefore, maybe white-collar employees’ levels of burnout and work performance are independent of the knowledge characteristics because these workers must deal with the knowledge complexity of work all the time, and they must rely on alternative support from WD to manage this source of burnout and to find ways to perform better.

These findings had some theoretical and practical implications for future research in the field and for organizational management. First, by uncovering the mediating roles of WF conflict and WF enrichment, the results showed the WF mechanisms through which some WD characteristics were related to burnout and work performance. Theoretically, this evidence contributed to expanding the knowledge in the WF literature, as it showed how two different dynamics, active at the WF interface (i.e., conflict and enrichment), could concur to strengthen the role of WD as a crucial organizational practice, useful also for managing employees’ burnout and performance. Practically, this study has given important inputs to organizations and managers on how to intervene in WD to reach the desired outcomes in terms of well-being and performance supporting the integration of work and family life. In this vein, the interventions of job redesign could foster work performance and prevent burnout operating on job demands and resources. For example, interventions at the organizational level should aim to optimize job demands and increase job resources by redesigning the work environment to provide more opportunities for relational exchanges [83]. Consequently, the promotion of relational exchanges through the adjustment of the contextual and social characteristics of work could affect the balance between job demands (e.g., WF conflict) and resources (e.g., WF enrichment) to limit burnout and improve performance [84]. Also, training interventions aimed at promoting personal resources could have consequences on burnout and performance [83], if they carefully consider the WD of a specific job profile and the surrounding work environment [85].

Second, the different effects of the four WD dimensions on WF aspects suggested which characteristics of WD that could be more powerful in explaining the relationships with burnout and work performance via WF interface aspects. Theoretically, this evidence indicated that the task, social, and contextual characteristics of work should be accurately examined in the investigation of the interrelationships between work and family. Practically, it suggested the focus of work redesign interventions aimed at enhancing the enrichment or decreasing the conflict between work and family domains. If managers find out or suspect that white-collar employees have issues in the interrelationships between work and family domains (e.g., high WF conflict and low WF enrichment), they should intervene in redefining the specific dimensions of work design without a radical modification of the entire WD. For example, managers could bring targeted changes in contextual characteristics to decrease WF conflict and in social characteristics to foster WF enrichment. Such targeted changes could prevent burnout and promote work performance without redesigning all the characteristics of a job.

Third, the different results found in relation to the hypotheses about the WF variables suggested that a negative mechanism (i.e., WF conflict) and a positive mechanism (i.e., WF enrichment) may have different roles in the paths from WD to burnout and to work performance. Specifically, WF conflict could be a negative mechanism that explains the association between WD and undesirable outcomes (e.g., burnout), whereas WF enrichment could be a positive mechanism that explains the association between WD and desired outcomes (e.g., work performance). Theoretically, this evidence indicated that WF conflict is a main variable to examine in the work processes that may lead to burnout. On the other hand, WF enrichment had a key role in the process of explaining how a high-quality WD leads to better performance and less risk of burnout. Practically, the study suggested that HR interventions in the field of the WF interface need to carefully consider the crucial role of WF culture in promoting WF enrichment and buffering WF conflict. More specifically, by focusing on enrichment rather than conflict between domains, WF culture might contribute to developing shared assumptions, beliefs, and values that could concretely support the integration of an employee’s work and family lives, individual and organizational well-being, and work performance. To do so, diversity training interventions could play a crucial role in disseminating an unbiased representation of work and family roles, supporting HR managers in defining coherent WF balance strategies.

The present study has limitations to be considered when interpreting its results. First, the sample is not representative of all Italian workers because it is limited to white-collar employees. This limitation is particularly relevant in WD research because WD dimensions are not universal for white-collar employees and other categories of workers. Consequently, the results could be affected by the specific cohort of workers that has been studied. Thus, the findings cannot be generalized because workers with different roles and positions may have different perceptions of the WD, as well as different sources of burnout or criteria of work performance. This limitation could explain the non-significant relationships of the knowledge characteristics, because white-collar jobs are characterized by high levels of information processing and skill variety that may be constant and may not affect other work dynamics. Future research should recruit a sample that includes different professional roles and positions to assess the variability of the WD dimensions. The replication of the model proposed in the present study in a larger sample could be useful to compare the results between the groups of white-collar and blue-collar employees. Second, the use of self-report measures and the cross-sectional design may have caused shared method variance and an overestimation of some relationships. Thus, it cannot be argued that the observed relationships were necessarily causal. In particular, the lack of longitudinal data does not allow us to control for the temporal biases caused by the trends of changes in the fields of the WF interface and WD literature. Different participants might respond differently based on the direction in which trends are heading, which could influence research findings. For example, the COVID-19 pandemic has brought contextual technological transformations which have introduced changes in the WD of certain jobs and in the ways of balancing work and family lives, affecting burnout as a consequence [86]. Future research should consider the trends of changes by adopting a longitudinal design and involve different types of measurement (e.g., multiple informants, external observers, etc.) to further investigate the direction of the associations over time and gather both the subjective experiences of employees and external objective evaluations.

In conclusion, the present study contributed to a wider understanding of the role of WD as a HR and management practice that could be related to burnout and work performance. The findings showed the mediating role of WF conflict in the relationships between the task and contextual characteristics and burnout. Moreover, the findings highlighted the mediating role of WF enrichment in the relationships between the task and social characteristics and burnout and work performance. The implications of the present study may contribute to enriching knowledge on the consequences brought about by managerial decisions and choices when designing work practices, consequently guiding the evidence-based HR and organizational interventions of work redesign.

## Figures and Tables

**Table 1:** Descriptive statistics, Cronbach’s alpha coefficients, and Pearson’s correlations associated with the study variables.

Variables	1	2	3	4	5	6	7	8
1. Task characteristics	[0.84]							
2. Knowledge characteristics	0.43 ***	[0.89]						
3. Social characteristics	0.58 ***	0.29 ***	[0.77]					
4. Contextual characteristics	0.00	−0.06	0.06	[0.72]				
5. WF conflict	−0.20 *	0.05	−0.11	−0.29 ***	[0.88]			
6. WF enrichment	0.55 ***	0.15	0.50 ***	0.07	−0.31 ***	[0.87]		
7. Burnout	−0.48 ***	−0.09	−0.37 ***	−0.18 *	0.44 ***	−0.60 ***	[0.89]	
8. Work performance	0.33 ***	0.19 *	0.31 ***	−0.08	−0.04	0.35 ***	−0.33 ***	[0.88]
Mean	33.05	25.17	22.13	23.34	27.73	20.86	24.96	39.05
Standard deviation	6.78	4.32	4.44	4.42	10.77	5.18	8.53	5.16
Skewness	−0.02	−0.48	−0.30	−0.47	0.25	−0.14	0.78	−0.72
Kurtosis	−0.56	−0.97	−0.12	−0.44	−0.62	−0.32	0.63	−0.21

Note. * *p* < 0.05; *** *p* < 0.001. Cronbach’s alpha coefficients are reported on the diagonal within brackets.

**Table 2:** Indirect effects of WD dimensions (i.e., task, knowledge, social, and contextual characteristics) on burnout and work performance via WF interface aspects (i.e., WF conflict and WF enrichment).

Indirect Effect	Estimate	SE	95% CI Lower	95% CI Upper
Task c. → WF conflict → Burnout	−0.10	0.04	−0.20	−0.03
Task c. → WF enrichment → Burnout	−0.23	0.07	−0.40	−0.11
Task c. → WF conflict → Work performance	−0.02	0.02	−0.06	0.01
Task c. → WF enrichment → Work performance	0.13	0.04	0.06	0.22
Knowledge c. → WF conflict → Burnout	0.08	0.05	−0.01	0.18
Knowledge c. → WF enrichment → Burnout	0.09	0.06	−0.01	0.24
Knowledge c. → WF conflict → Work performance	0.01	0.02	−0.01	0.07
Knowledge c. → WF enrichment → Work performance	−0.05	0.03	−0.13	0.01
Social c. → WF conflict → Burnout	0.01	0.05	−0.08	0.11
Social c. → WF enrichment → Burnout	−0.22	0.08	−0.42	−0.09
Social c. → WF conflict → Work performance	0.00	0.01	−0.01	0.04
Social c. → WF enrichment → Work performance	0.12	0.05	0.04	0.23
Contextual c. → WF conflict → Burnout	−0.15	0.06	−0.29	−0.05
Contextual c. → WF enrichment → Burnout	−0.04	0.06	−0.16	0.06
Contextual c. → WF conflict → Work performance	−0.03	0.03	−0.10	0.02
Contextual c. → WF enrichment → Work performance	0.02	0.03	−0.03	0.08

Note. Parameter estimates are unstandardized coefficients. SE—standard error; CI—confidence intervals; task c.—task characteristics; knowledge c.—knowledge characteristics; social c.—social characteristics; contextual c.—contextual characteristics.

## Data Availability

The data presented in this study are available on request from the corresponding author. The data are not publicly available due to privacy restrictions.
